# Comparison of the morphology of the anterior cruciate ligament and related bony structures between pigs and humans

**DOI:** 10.3389/fvets.2022.1045785

**Published:** 2022-11-18

**Authors:** Qinyi Shi, Huizhi Wang, Kaixin He, Mingzhu Tao, Cheng-Kung Cheng

**Affiliations:** Engineering Research Center of Digital Medicine, School of Biomedical Engineering and Med-X Research Institute, Ministry of Education, Shanghai Jiao Tong University, Shanghai, China

**Keywords:** anterior cruciate ligament (ACL), pig, joint anatomy, knee morphology, comparative medicine

## Abstract

**Introduction:**

Pigs are widely used for clinical research on the anterior cruciate ligament (ACL) because of the similarity of the knee structure to the human knee. But evidence to support the suitability of using porcine samples to guide clinical practices is limited. This study aims to explore the qualitative and quantitative morphological features of the porcine knee and ACL, and to compare these with data on humans reported in literature.

**Methods:**

Nineteen porcine knees were used for this study. The bone structures were measured on coronal X-ray images. The length of the ACL was measured using a caliper. The ACL bone insertion sites were marked and measured on a digital photograph. The lengths of the long and short axis of the ACL isthmus were measured on the X-ray microscopy reconstructed images. The outcomes were compared with previously reported data on humans using an abstract independent-samples *T* test.

**Results:**

Qualitative observation indicated a similar location, orientation and general morphology of the porcine ACL to human ACLs. The major difference was the location of the ACL tibial insertion with respect to the anterior horn of the lateral meniscus (AHLM). The porcine ACL was split into AM and PL bundles by the AHLM, while the AHLM was adjacent to the anterolateral border of the ACL tibial insertion in human knees. The quantitative comparison showed no significant difference between the human and porcine ACL in terms of the length of the ACL, the width of the femoral condyle and tibial plateau, and the tibial interspinal width. However, the CSA, the lengths of the long and short axis of the ACL isthmus, and the femoral and tibial insertion areas of the porcine ACL were all significantly larger than the reported features in human knees.

**Conclusion:**

The location, orientation and basic morphology of the porcine ACL and knee are similar to humans. However, the two-bundle structure is more distinct in a porcine ACL, and the dimensions of the porcine ACL are generally larger. This study may provide useful information to researchers when assessing the feasibility and limitations of using porcine samples for research on the human ACL and knee.

## Introduction

The anterior cruciate ligament (ACL) is an important ligament in the knee joint for maintaining the stability of articular movement (1). However, rupture of the ACL is increasingly common, probably due to the increasing popularization of sports. Research on the anatomy and biomechanical function of the ACL is beneficial for preventing injury and improving clinical treatment.

*In vivo* human studies are often more difficult to perform than *in vitro* laboratory research using cadaveric knees, mainly because of the invasive methods required and considerable ethical constraints. The acquisition of human cadaveric samples for performing *in vitro* studies is also restricted for both ethical reasons and a scarcity of samples. In this context, animal samples have become a popular substitute, among which pigs are one of the most widely used species for exploring clinical problems related to the ACL. Proper use of porcine samples can reduce the research budget and ease the complicated process of applying for ethical approval. Porcine knees have been used extensively in biomechanical research to understand the role of the ACL in knee functionality (2), constraining the knee (3), to investigate ACL healing after surgery (4–6), and the microstructure of ACL enthesis (7).

Among the aforementioned studies, only a few discussed the suitability of using porcine samples for guiding clinical practice on humans. Proffen et al. (8) compared the general anatomy of the intra-articular structures between humans and six animal species (cow, sheep, goat, dog, pig, and rabbit). The tibial insertion of the human ACL is adjacent to the AHLM, but for the porcine knee, the AM and PL bundles of the ACL are split by the ALM (8–10). Porcine knees also have a longer ACL than humans, and thus are not suggested as the best choice for ACL research. In contrast, Bascuán et al. (11) assessed the anatomy and biomechanics of the ACL in five large laboratory animals and compared those against the human ACL. Bascuán concluded that goats and pigs were most similar to humans in terms of the number of bundles, location of insertion sites, vascular supply, topography of the tibial plateau, mechanical function and ultimate load. Xerogeanes et al. (10) measured the *in situ* force of the ACL in humans, goats, pigs, and sheep and found that porcine knees may be the most suitable for mechanical studies on the human ACL. However, in contrast to the two bundles reported by other investigators, Kato et al. (2) divided the porcine ACL into three bundles and tested *in situ* forces in each bundle and concluded that the AM and PL bundles of the porcine ACL have similar roles to the corresponding bundles in the human knee.

These studies and related publications have improved knowledge on the mechanical function and morphology of the porcine knee and ACL and discussed similarities and differences to the function of the human ACL. However, controversy remains around the number of bundles and location of the bone insertions for the porcine ACL. There is a lack of comprehensive and quantitative measurements of the three-dimensional morphology of the porcine ACL and related bony structures, which is important for understanding its biomechanical role and the suitability as a substitute for research on the human ACL. The purpose of this study was to qualitatively and quantitatively explore the morphological features of the porcine ACL and related bony structures in the knee, including the widths of femoral and tibial condyles, width and height of the femoral notch, width of the tibial interspinal fossa, location and areas of ACL bone insertion sites, shape of the ACL and its bone insertion sites, and cross-sectional area of the ACL isthmus and ACL length. The recorded measurements would be compared to data for the human knee from literature. It was hypothesized that all of the measured morphological parameters of the porcine knees were within the range of the human knee, demonstrating the suitability of porcine models for research on the anatomy and function of the human knee and ACL. This study may help with research planning to determine the feasibility and clinical suitability of using porcine samples for conducting scientific research on the anatomy, biomechanics and pathologies of the human ACL.

## Materials and methods

### Samples

The research protocol was approved by the university's Institutional Animal Care and Use Committee. Nineteen porcine knees (Duroc-landrace-yorkshire crossbred, male, and 1 year) were harvested from a licensed slaughterhouse and preserved at −20°C at our laboratory within 24 hours of slaughter. The samples were thawed at room temperature for 12 hours prior to use. Soft tissues were removed approximately 12 cm proximal and distal to the joint line and the samples were kept hydrated in gauze wetted with saline. Previous studies measured the dimensions of the human knee joint from anteroposterior X-ray images (12–15). As such, anteroposterior X-ray images were taken of the porcine knees to determine the corresponding morphological parameters of bony structures.

### Measurement of bone structures in porcine knee joint from anteroposterior X-ray images

**Figure 1:**
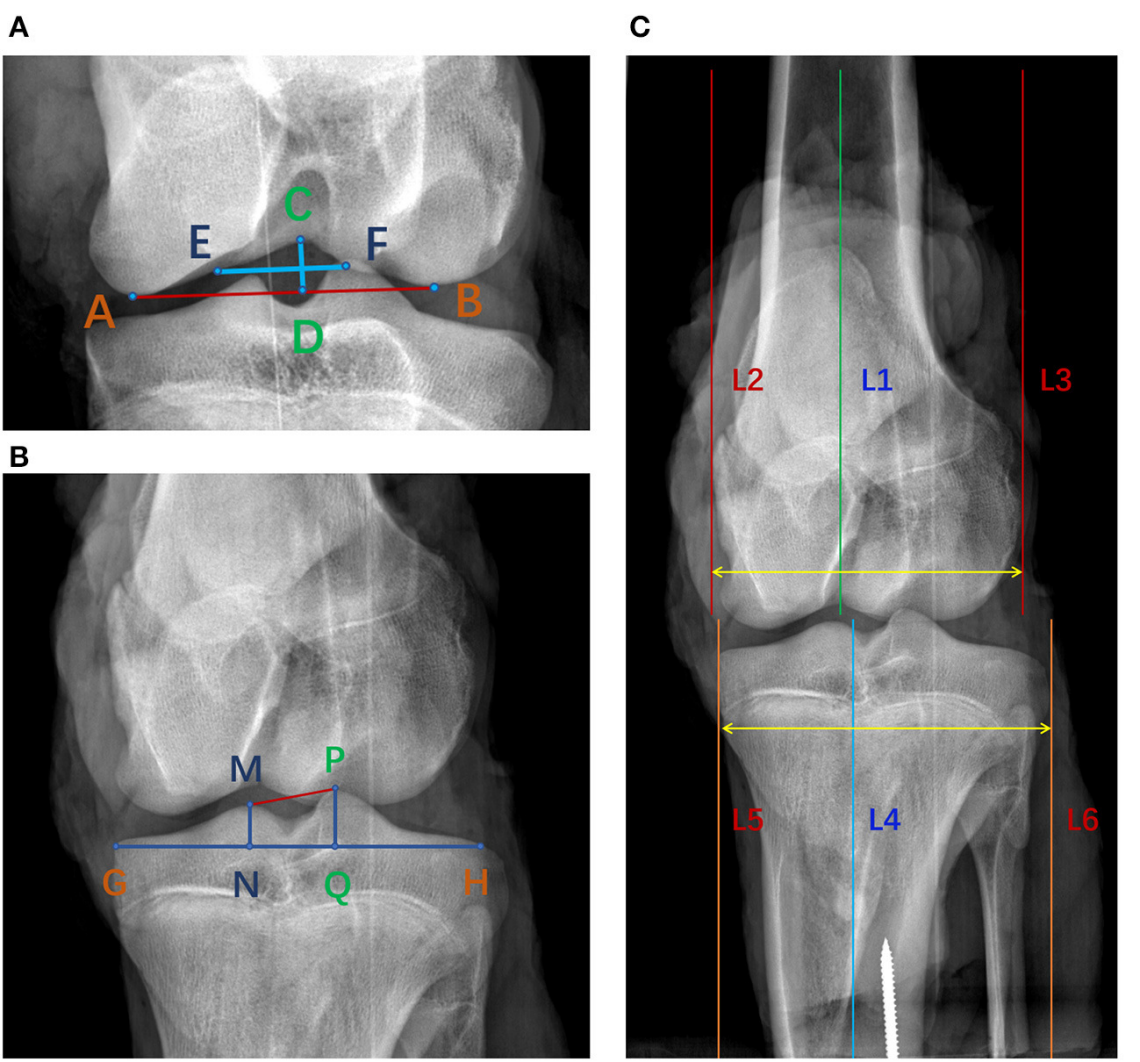
Measurement of bony parameters on coronal radiographs of porcine knee. **(A)** Line CD was the notch height and line EF was the notch width. S1 was the area of the femoral notch. **(B)** Line NQ was the width of the tibial interspinal fossa. S2 was the area of the tibial interspinal fossa. **(C)** The distance between line 2 and line 3 was the width of the femoral condyle and the distance between line 5 and line 6 was the width of the tibial condyle.

As shown in Figure 1, the most distal points of the medial and lateral femoral condyles were defined as point A and point B, respectively. Point C was the most proximal point of the femoral notch. A line perpendicular to line AB passing through point C was defined as the notch height (length of line CD). Line EF was perpendicular line CD and passed through its center, and defined the notch width (12). Similar to the femoral notch, line GH was plotted tangent to the tibial plateau. The vertex of the tibial ridges was defined as points M and *P*. The perpendiculars to the line GH passing through points M and *P* respectively intersected GH at point N and point Q. The line NQ was defined as the width of tibial interspinal fossa (13). In Figure 1C, lines 1 and 4 were the anatomical axes of the femoral and tibial shafts. Lines 2 and 3, plotted parallel to line 1, were the medial and lateral margins of the femoral condyle. Lines 5 and 6, parallel to line 4, were the medial and lateral margins of the tibial condyle. The distance between line 2 and line 3 was defined as the width of the femoral condyle and the distance between line 5 and line 6 was defined as the width of the tibial condyle (14, 15).

### Qualitative observation of the macro-morphology of the porcine ACL

The porcine knee joints were fixed at full extension in 10% formalin at room temperature for 48 hours, after which the patella and soft tissues surrounding the knee were removed to expose the intracapsular structures for qualitative observation. The removal of soft tissues was performed by one of the authors who is familiar with the standard process to expose the ACL and performed several similar studies in the past. Next, the medial femoral condyle and the menisci were carefully removed to allow for a better viewing of the whole ACL. During the dissection, the relative position, macromorphology and connectivity of the ACL with adjacent tissues were observed. Specifically, the shape of the ACL, location of its bone insertions, the general size of the ACL relative to the femoral notch and condyles, the number of ACL bundles, and the mode of twisting of the bundles were observed for further comparison with those from human ACLs published in literature.

### Measurement of morphological parameters of the ACL

**Figure 2:**
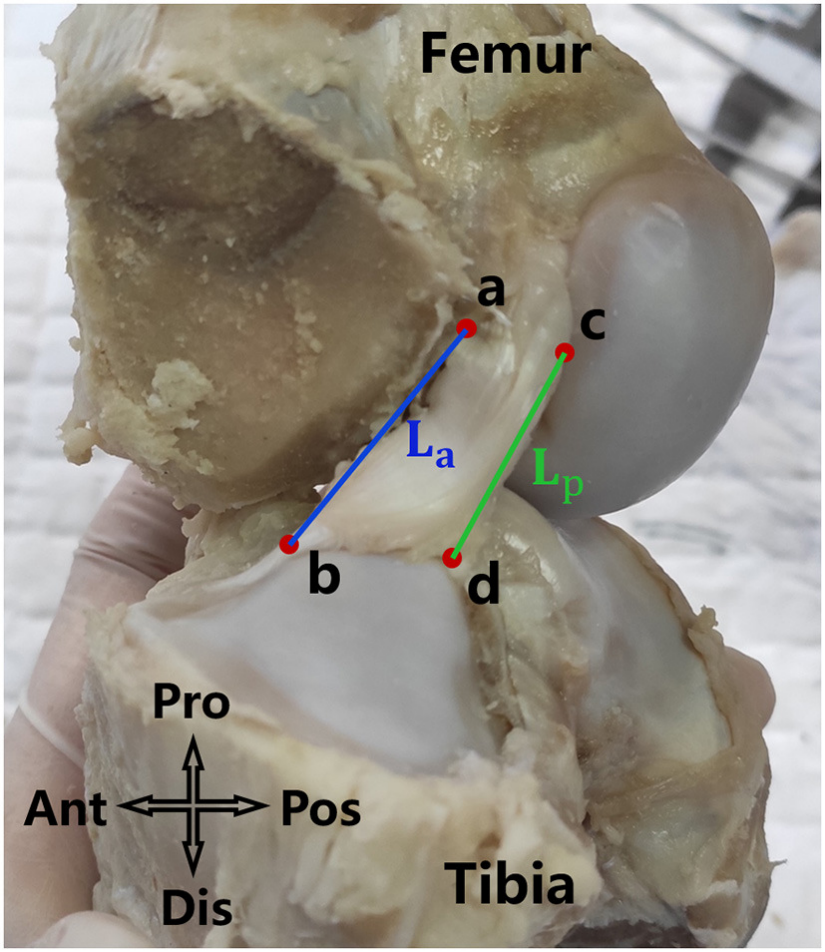
La and Lp were respectively the anterior and posterior length of the ACL. The average of *La* and *Lp* was defined as the length of the ACL.

With the knee in full extension, an electrical caliper with an accuracy of 0.01 mm was used to measure the length of the ACL in a sagittal view. As shown in Figure 2, points a and c were the most distal points of the femoral origin of the ACL and points b and d were the most proximal points of the tibial insertion of the ACL. *La* and *Lp* represent the anterior and posterior lengths of the ACL, and were averaged to represent the effective length of the whole ACL (Figure 2).

**Figure 3:**
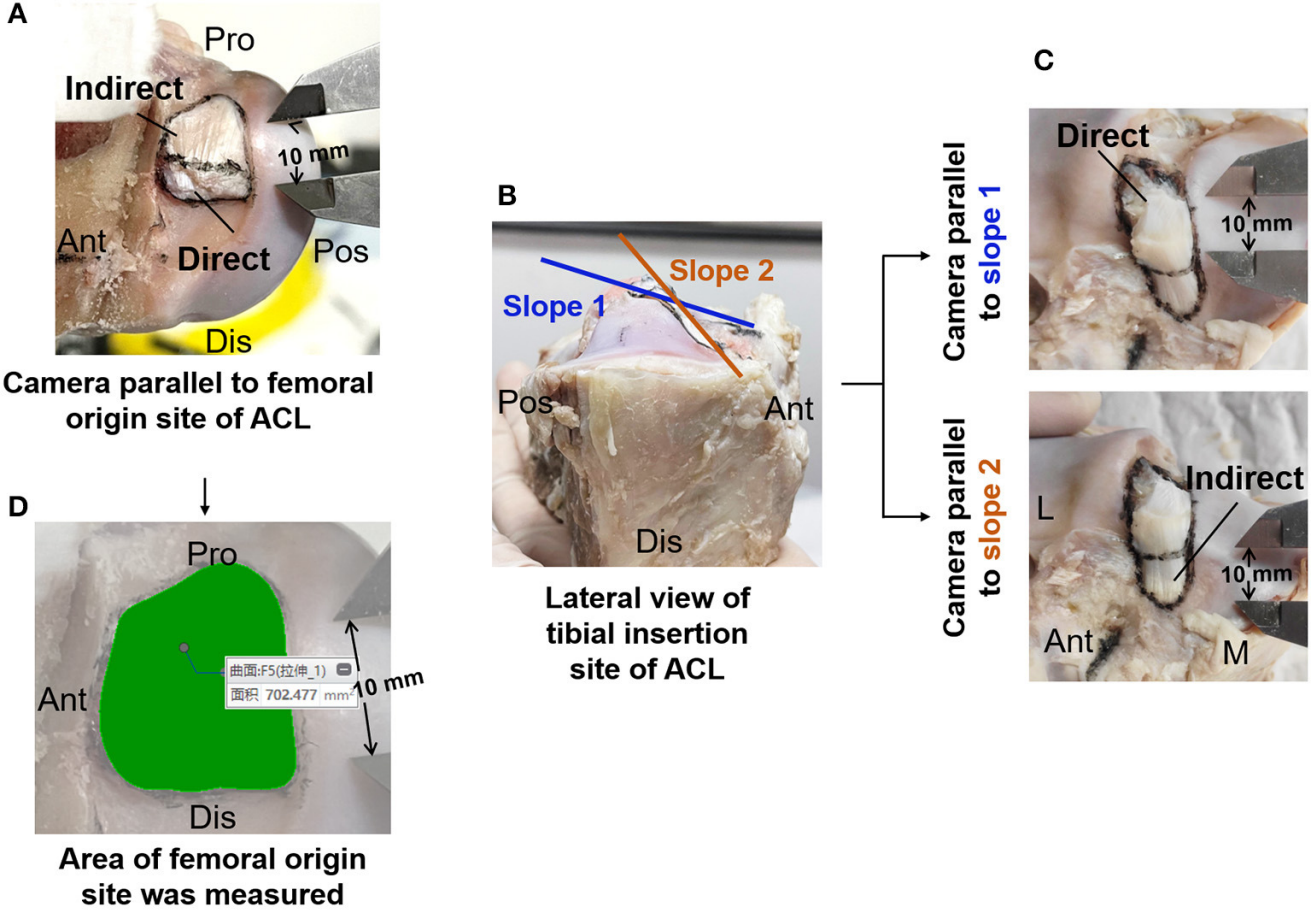
Measurement of insertion site of ACL. **(A)** Image of femoral origin site of ACL divided into direct and indirect part. **(B)** Double-sloped tibial insertion site of ACL with the slopes shown parallel to the direct and indirect part. **(C)** Image of tibial insertion site of ACL. **(D)** Area of femoral origin site measured in Creo Parametric 7.0 software (PTC, Massachusetts, USA) (precision 0.001 mm). M, medial; L, lateral; Ant, anterior; Pos, posterior; Pro, proximal; Dis, distal.

Then, the ACL substance was cut off along its femoral and tibial bone insertion sites. The circumferences of the femoral and tibial insertion sites were circumscribed with a marker pen which was further divided into direct and indirect parts according to whether the ligament had a direct connection to the mid-substance fibers of the ACL (Figures 3A,C). The indirect part is not directly attached to the mid-substance fibers of the ACL but is a broader fan-like extension of the direct part. Digital photographs were taken to obtain the largest projected area of the marked regions which represent the best approximation of the surface area of the insertion sites. While photographing, a vernier caliper was placed near the circumferences of the insertion sites to allow the measurements to be calibrated. The total area and the areas of the direct and indirect insertion sites of the ACL were respectively measured on the photographs using Creo Parametric 7.0 (PTC, Massachusetts, USA) (precision 0.001 mm) (Figure 3).

**Figure 4:**
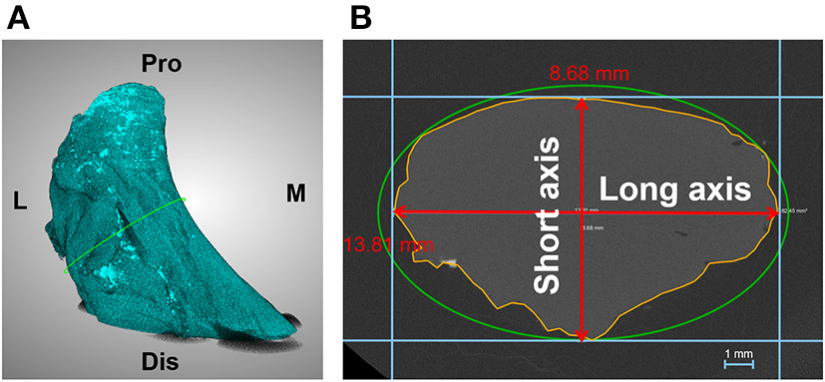
Measurement of dimensions of the ACL isthmus. **(A)** ACL substance reconstructed in Dragonfly. **(B)** Cross sectional area and long axis and short axis of the ACL isthmus (enclosed by yellow line) was measured on the isthmus plane. M, medial; L, lateral; Pro, proximal; Dis, distal.

The ACL substances were enveloped with plastic film and scanned by X-ray microscopy (Zeiss Xradia 520 Versa, Carl Zeiss AG, Germany) at a resolution of 30 × 30 × 30 μm to acquire the accurate morphology of the ACL. One thousand one hundred one projections were measured with an exposure of 1 s at 70 kV and 6 W. The sample-source distance was 81.8 mm and the sample-detector distance was 104.0 mm. The projected images were imported into Dragonfly software (ORS, Montréal, Canada) to measure the cross-sectional area (CSA) and the lengths of the long and short axis of the ACL isthmus (Figure 4). Firstly, a cross-sectional plane was defined to be perpendicular to the isthmus of the ACL. The CSA was measured on this plane with the freehand shape tool in Dragonfly. To measure the long axis and short axis of the ACL, a circumscribed ellipse of the CSA and its long and short axis was drawn. Then, lines were drawn parallel to the long and short axis of the ellipse and tangent to the border of the ACL isthmus on the plane. The length of the lines between the parallel lines were respectively defined as the long axis and short axis of the ACL isthmus.

All measurements were conducted by two independent observers, and the first observer measured twice. The intra- and inter- observer reliabilities were calculated using the intraclass correlation coefficient (ICC) with a 2-way random model of absolute agreement. The ICC can assume any value from 0 to 1, where a value greater than 0.80 represents good agreement, between 0.60 and 0.79 signifies moderate agreement, and 0.59 signifies poor agreement.

### Comparison of morphological features of the porcine ACL and knee joint to human

Qualitative observation and quantitative measurements on the porcine knee and ACL were compared with those previously reported for humans (12, 13, 16–20). Related publications were searched through the Web of Science with the following search terms: ACL, insertion, attachment, knee, tibial spine, femoral notch with measurement, morphology, anthropometry, shape, width, length, and height. Those published from 2000 to 2020 were selected. The most recent and highly cited publications which used similar measurement methods to this study were preferentially referred to for the comparison. According to how results were presented in the previous studies, the average values, the values of standard deviation, or the minimum and maximum values for the measurements were correspondingly chosen to facilitate the comparison. The location, shape, and morphological characteristics of the porcine ACLs and the bone insertion points were compared against the human ACL data (12, 13, 16–20). The morphological parameters recorded in this current study, including the ACL length, cross-sectional area of ACL, area of bone insertion points, width of femoral and tibial condyles, and the width and area of the femoral notch and tibial spines, were compared with the corresponding measurements reported in previous studies. According to the outcome of the comparison, the suitability of using the porcine model for different types of studies on the human knee and ACL were analyzed and discussed.

### Statistical analysis

All statistical analyses were performed in IBM SPSS Statistics 25 (SPSS Inc, Chicago, USA). A single sample Kolmogorov-Smirnov test was used to ensure that all the measurement results were normally distributed and the sample size was sufficient. An abstract independent-samples *T*-test was used to evaluate whether the recorded parameters for the porcine knee and ACL were significantly different to the reported human data. Equal variances were not assumed. Statistical significance was assumed when *P* < 0.05 for the Kolmogorov-Smirnov test and *P* < 0.01 for the abstract independent-samples *T*-test.

## Results

### Macroscopic comparison of porcine ACL to human ACL

Through dissection, the macroscopic shape of the porcine ACL and its relative position to the knee were observed. In the knee joint, synovial membranes connect adjacent fibrous connective tissues to form a semifixed structure. Therefore, when cutting the posterior cruciate ligament, it is important to be mindful not to damage of the ACL.

**Figure 5:**
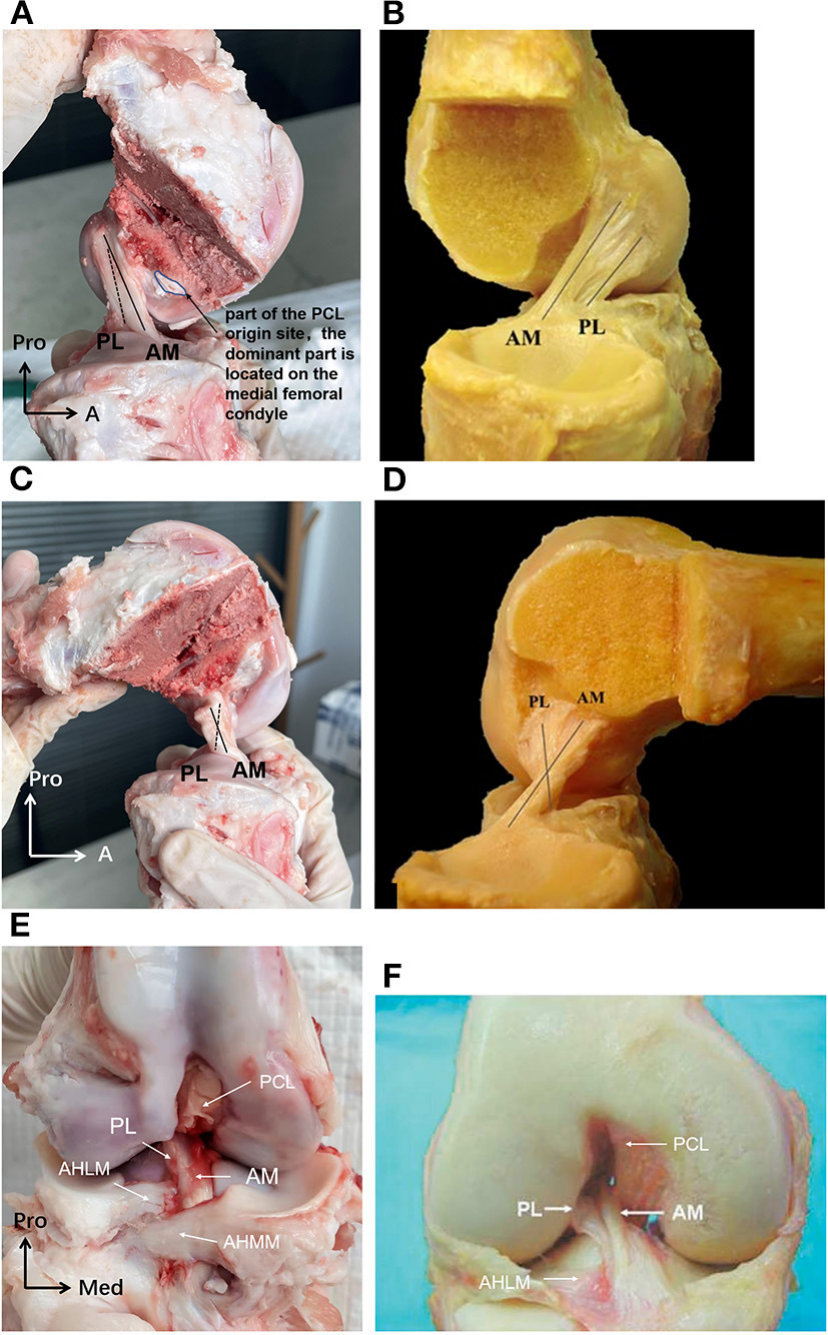
**(A)** The porcine ACL at full knee extension in sagittal view. The AM bundle was lax while the PL bundle was tight, and the two bundles were almost parallel. **(B)** The human ACL at full knee extension in sagittal view. **(C)** The porcine ACL with knee flexed to around 90. The AM bundle was tight while the PL bundle was lax, and the two bundles formed an intersecting structure. **(D)** The human ACL with knee flexed to around 90. **(E)** The porcine ACL in the coronal plane. The AM bundle of the porcine ACL was anterior and medial to the PL bundle at the tibial insertion. The AHLM (anterior horn of the lateral meniscus) split the porcine ACL into AM and PL bundles. **(F)** The human ACL in the coronal plane. The AM bundle of the human ACL was anterior and medial to the PL bundle at the tibial insertion. The AHLM borders on the lateral side of the human ACL (23).

**Figure 6:**
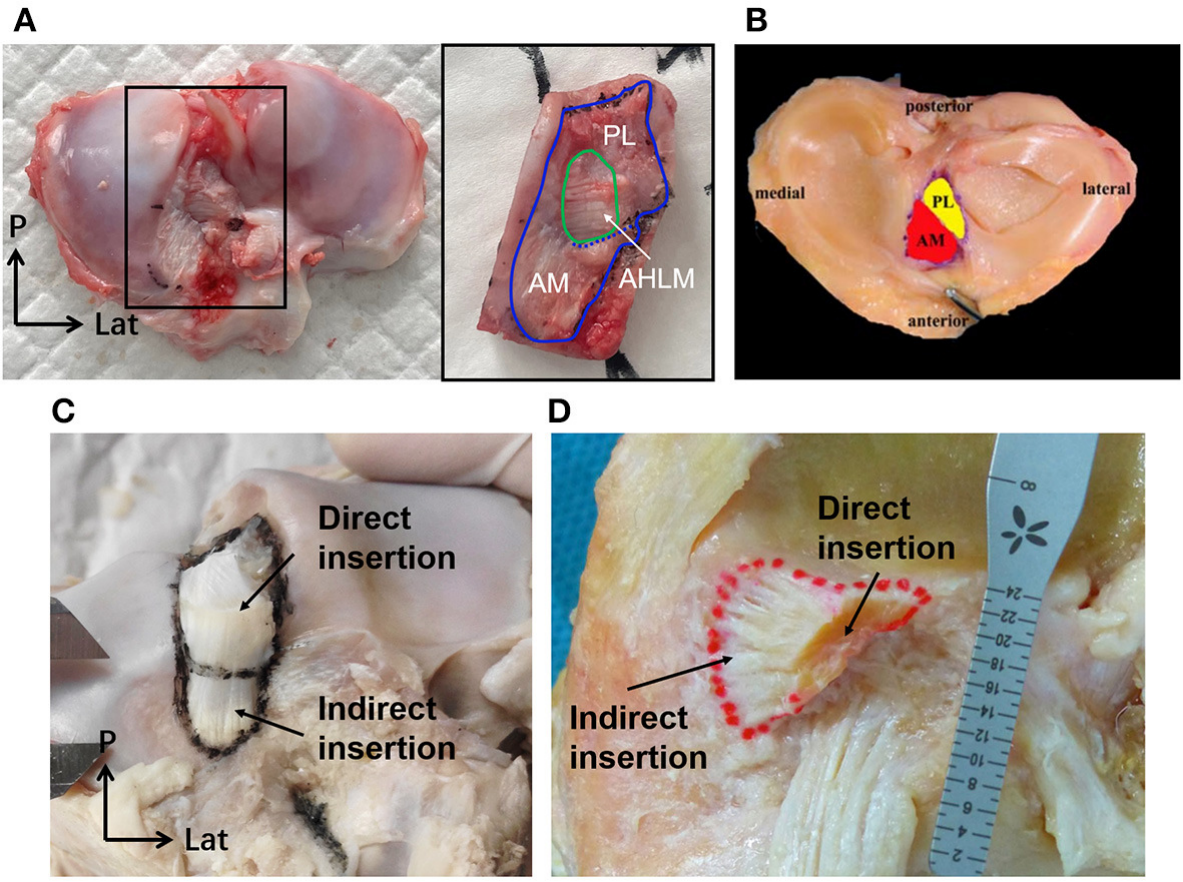
The tibial insertion of porcine and human ACLs. **(A)** The AHLM split the porcine tibial insertion into AM and PL bundles. **(B)** The AM and PL bundle of the tibial insertion for a human ACL. **(C)** The direct and indirect tibial insertion pattern of a porcine ACL. **(D)** The direct and indirect tibial insertion pattern of a human ACL.

**Figure 7:**
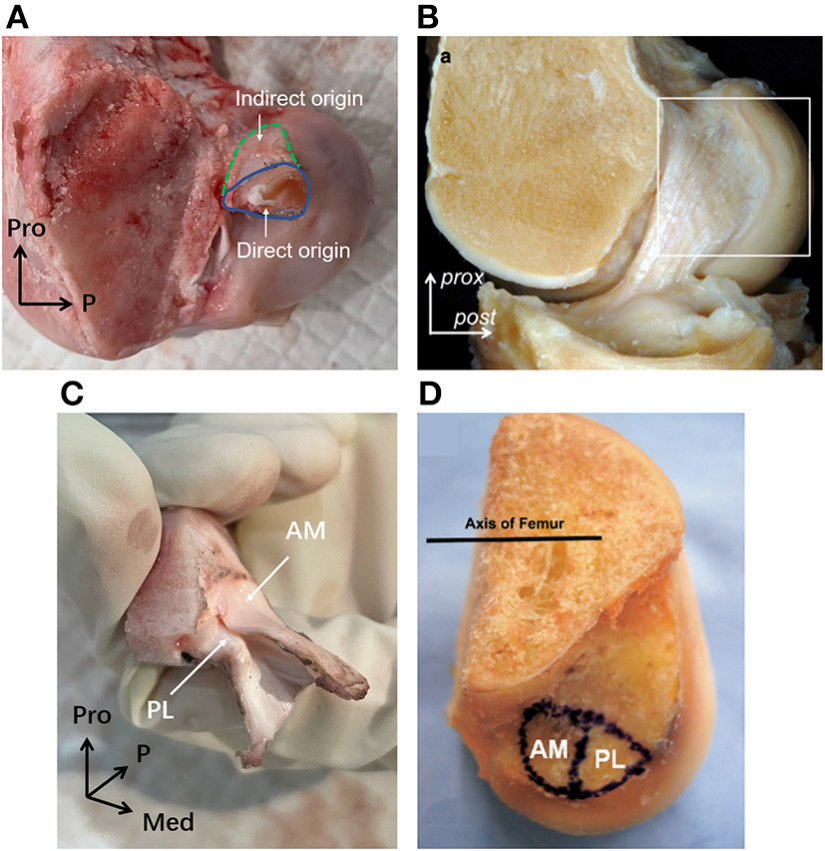
The femoral origin of porcine and human ACLs. **(A)** The direct and indirect origin regions of porcine femoral ACL insertion. **(B)** The mid-substance and fan-like extension of human ACL femoral origin. **(C)** The AM and PL bundle femoral origin of porcine ACL. **(D)** The AM and PL bundle femoral origin of human ACL.

At full knee extension, the porcine ACL extended from the medial side of the lateral condyle of the femur, then went medially, and inserted into the anterior edge of the tibial plateau (Figures 5A,C). It is known that the human ACL attaches lateral and anterior to the medial intercondylar spine of the tibia, and to the medial aspect of the lateral femoral condyle (21–23) (Figures 5B,D,F). The femoral origin of the porcine ACL was located on the posterosuperior part of the medial side of the lateral condyle of the femur, with its posterior border clinging to the posterior articular margin of the lateral femoral condyle and its anterior border clinging to the lateral intercondylar ridge. The femoral origin of the human ACL is generally described as traveling obliquely from anteroproximal to posterodistal and is located on the posterior part of the medial surface of the lateral condyle (18, 21, 24) (Figures 5, **7**). The porcine tibial insertion was located on the anterior medial section of the tibial spines and extended anteriorly to form an indirect part (Figures 5A,C,E, 6A,C). The human tibial insertion is described as beginning behind the anterior border of the tibia and extending to the medial and lateral tibial spine (21, 22, 25) (Figures 5B,D,F, 6B,D). Therefore, the gross anatomical location of the ACL bone insertions is similar in pigs and humans.

The porcine ACL presented as an hourglass shape with fan-like insertions on the femur and tibia (Figures 5A,C,E). The ACL substance appeared as a ribbon shape rather than column. After removing the synovial membranes that wrapped the ACL fibers, a distinct double-bundle structure could be observed. One bundle was anterior and medial to the other bundle at the tibial insertion and was proximal and posterior to the other bundle at the femoral origin (Figures 5A,C). Correspondingly, the human ACL is widely reported as being divided into anteromedial (AM) and posterolateral (PL) fiber bundles (21–23, 26). In addition, the AM bundle of the porcine ACL was longer and wider than the PL bundle. When the knee was extended, the two bundles were almost parallel (Figure 5A). When the knee was flexed, the AM bundle which was originally at the proximal femoral origin turned posteriorly, thus forming an intersecting structure with the PL bundle (Figure 5C). These changes in orientation of the AM and PL bundles in the porcine ACL were similar to the orientations reported for human ACLs (21–23, 26) (Figure 5).

Part of the bone insertion of the porcine ACL was directly connected to the mobile mid-substance fibers of the ACL (called direct insertion, Figures 6C, 7A), while the other part was an extended fan-like section from the direct part (called indirect insertion, Figures 6C, 7A) and attached to the bone at a sharp angle. Fibers in the direct insertion were flexible in changing directions along with the sway of the mid-substance fibers, while the fibers in the indirect insertion were relatively immobile.

The tibial insertion had a cashew shaped attachment to the anterior medial section of the tibial spines and extended along the anterior part of the medial tibial spine. The AM bundle was anterior to the medial tibial spine and extended to the anterior border of the tibia, while the indirect insertion of the PL bundle lay beneath the AM bundle and extended posterior and lateral to the tibial spine. As shown in Figure 6A, in the porcine knee, the anterior horn of the lateral meniscus (AHLM) split the ACL into AM and PL bundles on the lateral side. But the two bundles were still connected to the medial side, making the whole structure appear like a folded page. The indirect insertion of the PL bundle was covered by the posterior half of the AHLM, and the anterior edge of the AHLM marked the boundary of the direct and indirect insertion of the AM bundle. Indirect insertion of the AM bundle was covered by the anterior horn of the medial meniscus (Figure 5E).

In humans, most tibial insertions have an oval shape. The anterior edge of the tibial insertion is covered by the transverse meniscal ligament. And the AHLM is adjacent to the anterolateral border of the ACL tibial insertion. In human knees, only a few fibers of the ACL are blended with the anterior attachment of the AHLM as well as with the posterior attachment of the posterior horn of the lateral meniscus (18). The anterior edge of the AHLM is also identifiable to distinguish the AM and PL bundles. However, the AHLM doesn't spilt the ACL in human knees, which appears to be different to the porcine ACL (Figures 2B, 5F). In humans. the ACL indirect tibial insertion extends from the direct insertion site anteriorly and broadly spreads toward the anterior rim of the tibial plateau (17) (Figure 6D).

As shown in Figure 7A, the porcine ACL femoral origin had an open umbrella-shaped insertion and was located posterior and proximal to the PCL origin (Figures 5A,E). The AM bundle lay proximal to the PL bundle. But the two bundles were only split at the anterior portion and merged at the posterior (Figure 7C). The direct origin was raindrop-shaped at the distal side of the femoral origin and the indirect part was the proximal extension (Figure 7A). For human ACLs, the AM bundle is more anterior than the PL bundle, and the indirect origin is on the proximal and posterior side of the femoral origin and borders on the margin of the articular cartilage (18) (Figures 7B,D).

From macroscopic observation, the major difference between the porcine and human ACL is that the AHLM splits the porcine ACL into AM and PL bundles on the lateral side at the tibial attachment. However, the porcine ACL is similar to the human ACL in terms of overall shape and positioning of the ACL and its insertions.

### Quantitative comparison of dimensions of the ACL and bony structures between porcine and human knees

Dimensions of the ACL and bony structures from the porcine knees in this study and those from human knees reported in literature are presented in Table 1.

**Table 1:** Quantitative comparison of the ACL and bone features in porcine and human knees.

	**Porcine**		**Human**			***N***	***P***	**Acquisition method**
	**Mean ±sd**	**Range**	**Mean ±sd**	**Range**	**Study**			
Length (mm)	26.1 ± 3.5	21.0–35.9	29.4 ± 4.7	22.2–36.5	Hashemi et al. (16)	15	0.032	Calipers
CSA (mm^2^)	67.4 ± 10.4	44.7–87.2	38.7 ± 7.7	20.3–51.5	Siebold et al. (17)	20	0	Digital camera and calipers
Long axis (mm)	12.8 ± 1.9	10.2–18.1	9.9 ± 1.5	7.0–12.7			0	
Short axis (mm)	7.5 ± 1.0	5.4–9.2	3.9 ± 0.7	2.8–4.9			0	
F-total (mm^2^)	227.7 ± 22.4	188.1–258.4	142.2 ± 24.4	110.5–188.8	Mochizuki et al. (18)	24	0	Digital photograph
F-direct (mm^2^)	138.7 ± 24.9	92.92–177.56	91.4 ± 23.7	54.3–142.3			0	
F-indirect (mm^2^)	88.9 ± 16.7	66.3–127.7	50.8 ± 12.6	28.8–74.4			0	
T-total (mm^2^)	301.2 ± 48.1	233.6–412.0	110.9 ± 14.7	80.1–133.1	Siebold et al. (17)	20	0	Digital photograph and calipers
T-direct (mm^2^)	114.3 ± 37.9	65.1–214.6	31.4 ± 7.2	18.5–45.0			0	
T-indirect (mm^2^)	186.9 ± 42.9	124.5–304.7	79.6 ± 12.7	53.7–107.7			0	
NH (mm)	6.94 ± 1.5	4.9–9.6	28.0 ± 3.1		Peng et al. (12)	67	0	X–ray
NW (mm)	14.4 ± 2.3	9.5–18.3	19.8 ± 2.4				0	
FW (mm)	69.9 ± 3.4	64.3–76.9	73.8 ± 3.9		Lipps et al. (19)	9	0.022	MRI
SW (mm)	13.9 ± 1.3	11.6–17.1	13.9 ± 2.1		Iriuchishima et al. (13)	37	1	X–ray
TW (mm)	72.9 ± 2.7	69.2–78.8	72.0 ± 6.4		Yahagi et al. (20)	39	0.455	Digital photograph and calipers

*N*, sample size; *Length*, ACL length; *CSA*, the cross sectional area of the ACL isthmus; *Long axis*, the long axis of the ACL isthmus; *Short axis*, the short axis of the ACL isthmus; *F-total*, area of total ACL femoral origin; *F-direct*, area of direct ACL femoral origin; *F-indirect*, area of indirect ACL femoral origin; *T-total*, area of total ACL tibial insertion; *T-direct*, area of direct ACL tibial insertion; *T-indirect*, area of indirect ACL tibial insertion; *NH*, the height of femoral intercondylar notch; *NW*, the width of femoral intercondylar notch; *FW*, the width of femoral condyle; *SW*, the width of tibial interspinal fossa; *TW*, width of the tibial condyle.

The results show that the difference in length between the porcine and human ACL is not statistically significant (*p* = 0.032). But the CSA of the porcine ACL (67.4 mm^2^) is significantly larger than the human ACL (38.7 mm^2^) (*p* = 0), and the long and short axis are both significantly longer in pigs (*p* = 0). The minimum value of the porcine short axis (5.4 mm) is longer than the maximum value of the ACL short axis in humans (4.9 mm). Similarly, it was found that the porcine ACL insertions are significantly larger than human ACL insertions on both the femoral and tibial side (*p* = 0). Particularly, the mean value of the total area of the porcine tibial insertion (301.2 mm^2^) is nearly triple that in humans (110.9 mm^2^).

Peng et al. (12) measured the height and width of the femoral notch on X-ray images of human knees with the knee flexed to 45°, while the current study on pigs recorded measurements at full knee extension. It was shown that the height and width of the porcine femoral notch (6.94 mm and 14.4 mm) are significantly smaller than humans (28.0 and 19.8 mm). However, there was no significant difference in femoral condyle width (69.9 vs. 73.8 mm), tibial interspinal width (13.9 vs. 13.9 mm), and tibial plateau width (72.9 vs. 72.0 mm) between the porcine and human knees (*p* = 0.022, 1, 0.455, respectively). In addition, the mean values of femoral condyle width, tibial interspinal width, and tibial plateau width for humans were all within the range of values from porcine knees in this study.

## Discussion

This study compared the macroscopic morphology of the ACL and related bone structures between pigs and humans to determine the suitability of using porcine samples for research on the knee joint and ACL. The qualitative observation indicated a high degree of similarity between the porcine and human ACL, including the overall shape and the position and orientation of the insertions relative to the femur and tibia. However, at the tibial attachment, the anterior horn of the lateral meniscus (AHLM) split the porcine ACL into AM and PL bundles. That was the most distinct difference in the gross anatomy of the porcine ACL compared to humans. The quantitative comparison showed that the CSA and the lengths of the long and short axis of the ACL isthmus, as well as the femoral and tibial insertion areas of the porcine ACL, were all significantly larger than a human ACL. However, there was no significant difference in the length of ACL, the width of the femoral condyle and tibial plateau, and the tibial interspinal width.

The gross anatomical location of the ACL bone insertions is similar between porcine and human knees, which is supported by earlier studies (8, 9, 27, 28). However, Fuss (9) compared the ACL of domestic pigs with humans and considered the femoral origin point in porcine knees to be more posteroproximal. This current study found the posterior border of the femoral origin of the porcine ACL to be tightly clung to the posterior articular margin of the lateral femoral condyle, which was similar to the arrangement depicted in human studies. The dispute over whether the porcine femoral origin is more proximal may be related to its larger area and differences in the definition of the boundary for the indirect region. The double bundle (AM and PL bundles) structure of porcine ACLs is consistent with the findings of Proffen et al. (8) on adult female Yorkshires pigs and Zhao et al. (7), who didn't mention the breed of pigs used. And the observation of the double bundle structure in pigs is similar to that previously reported for humans (29). However, Tantisricharoenkul et al. (27), Maeyama et al. (30), and Kato et al. (31) observed three bundles in the porcine ACL: anteromedial (AM) bundle, posterolateral (PL) bundle and intermediate (IM) bundle (Hampshire, 7–8 months). It is not known why some studies found two bundles while others reported three bundles, but the difference may be related to the breed of pigs used. Similarly, some studies have argued for three fiber bundles in the human ACL, but it is more widely accepted that the human ACL consists of two AM and PL bundles. Observation in this current study showed that the porcine ACL insertion can be divided into two parts, namely the direct insertion and indirect insertion. Yamauchi et al. (32) also observed in mature porcine knees that the anterior attachment of the femoral ACL site was a direct insertion and the posterior attachment could be characterized as an indirect insertion. This finding is consistent with the current study, and with previous studies on human knees (17, 18, 33). However, the indirect part of the porcine ACL femoral origin was located proximally on the knee while it was reported to be more posterior in humans. Nevertheless, this current study found that lines connecting the centers of the direct and indirect insertions in both porcine and human knees were aligned with the direction of ACL longitudinal fibers.

The finding that the AHLM separates the AM and PL bundles into two distinct bundles in porcine knees has also been reported in previous studies on pigs and other large mammals such as cows and sheep (8, 9). In humans, the AHLM is adjacent to the anterolateral border of the ACL tibial insertion and does not split the insertion. We suspect that this may be related to differences in gait between quadruped and bipedal mammals. Also, the mid-substance fibers in the porcine ACL can be further separated along the AHLM fissure through blunt dissection. However, it was observed that the porcine ACL cannot be totally separated into two independent bundles since the bundles are connected at the back, presenting like a folded page with the opening at the front. A membranous tissue between the two fiber bundles on the open side acts to bind them together to achieve cooperative but distinguishable functions. These features have not been reported in previous studies.

In terms of the quantitative results, the difference in length between the porcine and human ACL was not significant (*p* = 0.032), but the CSA of the mid-substance of the porcine ACL was significantly larger. In other words, the human ACL was thinner than the porcine ACL. This may be due to several reasons, one of which may be the human knee being more flexible in extension-flexion with the ability to move to 0° in extension, while the porcine knee can only extend to around 30° (8, 9). The valley in the femoral notch becomes shallower as the knee extends, meaning that the greater extension of the human knee may result in a smaller space for accommodating the ACL and so evolved to have a narrower CSA. In addition, the ACL in both bipedal and quadrupeds becomes tensed and more vulnerable during knee extension (34, 35), and mainly functions to prevent posterior sliding of the femur against the tibia (34, 35). Previous studies found that the anterior tibial load (which forces the femur to glide posteriorly against the tibia) during daily activities is proportional to the body weight (36), and thus the load on the ACL would also depend on the body weight. Considering that pigs are more massive (200–240 kg) than humans, the larger ACL may have evolved to resist these forces.

The results also showed a wide range of values for the CSA of the mid-substance and insertion area for both the porcine and human samples. While the statistical analysis in this study shows that porcine ACLs are significantly larger than human ACLs, the crossover between the ranges for pigs and humans indicates that the dimensions of a specific porcine ACL may not be larger than the native human ACL. Accordingly, studies should consider how individual differences in the size of the ACL may affect the outcome and whether a specific porcine ACL is representative.

The shape of the femoral and tibial insertions of the porcine ACL were similar to insertions reported for human subjects in literature. Most human tibial insertions were reported to have an oval shape, but there are also triangular or “C”-shaped types (37). This current study found that the tibial insertion of the porcine ACL has a cashew shape when the AHLM inserts medially. This was similar to the reported “C” shape in human knees (37, 38). On the other side, the femoral origin of human ACLs is commonly described as having a crescent or ribbon shape (39). This is similar to the porcine ACL femoral origin, but the porcine samples were thicker in the proximal-distal direction and had an open umbrella-shaped area that may be related to the pigs having an overall thicker ACL. The indirect part of the porcine femoral origin was mainly proximal while the indirect part of the human femoral origin was further posterior. However, lines connecting the centers of the direct and indirect insertions followed the direction of the ACL longitudinal fibers for both humans and pigs. This unique feature may facilitate the fibers in the direct insertion to move easily in flexion-extension but keep the fibers in the indirect area firmly fixed to resist tangential forces along the fiber direction. The different direction of longitudinal fibers in the human and porcine ACL may be a result of the different physiological knee flexion angles. Porcine knees cannot fully extend and are limited to a physiological extension angle of about 30° (8, 9). Therefore, although the indirect part of the porcine femoral origin was proximal to the flexion-extension axis, it was still posterior and proximal to the knee joint at full extension, which is similar to the orientation in human knees when at full knee extension. This would result in a similar force direction in both human and porcine ACLs at the same knee flexion angle. In terms of size, the porcine ACL insertion area is significantly larger than the human ACL insertion area, which is consistent with the larger mid-substance of the porcine ACL.

The height and width of the porcine femoral notch measured were significantly smaller than in human knees (12) when the human knee is flexed to 45°. The height of the femoral notch in human knees is almost four times greater than in pigs. Proffen et al. (8) also reported a narrower femoral notch in pigs than in humans, but the knee flexion angle at which the measurements were taken was not clearly described. We propose that it is possible the height and width of the porcine femoral notch are not necessarily smaller in pigs than in humans when measured at the same knee flexion angle. Greater knee flexion angles often result in a larger projected height and width of the femoral notch on the coronal plane, and thus flexing a human knee to 45° may show a larger projected dimension of the femoral notch than a porcine knee at full extension. This current study also did not measure the dimensions of the porcine femoral notch at the same knee flexion angle as the referenced literature for human knees because there is no scientific criteria for matching the flexion angle of porcine knees to humans. The results showed no significant difference between the porcine and human knees in terms of the width of the femoral condyle, width of the tibial plateau, and the tibial interspinal width. Thus, in general, the dimensions of the porcine knee are similar to human knees, except for the size of the femoral notch. The results of this study suggest that when porcine knees are used to represent human knees for scientific research, differences in dimensional features may have a negligible effect on the outcome.

Given the similarities and differences in the general shape and quantitative dimensions between the porcine and human ACL, researchers should carefully consider the feasibility of using porcine subjects, and there might be limitations to extrapolating the results to human applications. This study suggests that porcine samples may be suitable for studying the general biomechanical mechanisms of load transmission and for assessing functional differences between the two bundles of the ACL or two types of bone insertions (direct and indirect), since the general orientation, morphology and function are similar between porcine and human ACLs. However, considering the significant differences in ACL dimensions and the relative location of the bone insertion sites to the bone shaft, porcine subjects may not be suitable for studies investigating the size and positioning of the ACL. Future studies may consider exploring differences in the morphological and mechanical features between pigs and humans with respect to age, gender, and physical environment.

The main limitations of this study are (1) the human data from referenced literature used subjects of different ages, races, and sexes and that difference may influence the comparison results. However, these variables have been controlled as well as possible. Data from young, mature subjects were prioritized for comparison with the porcine subjects in the current study. (2) The measurement methods used in previous studies may not be identical to the methods used in this study, but any differences have been stated and discussed. (3) The largest projected area of the marked areas on the digital photographs was measured as the surface area of the insertion sites. However, the insertion is not flat, and thus the actual value may be slightly larger than the measured value. But this measurement method is same as that used in previous studies referenced, and so will have a negligible impact on the comparison results.

## Conclusion

This study found that the location, orientation, and general morphology of the porcine ACL and related bony structures in the knee joint are similar to humans. However, the two bundles in the porcine ACL were more distinguishable than in humans because they are split by the AHLM, and the dimensions of the ACL are generally larger in the porcine knee, except for the length of the ACL. The outcomes of this study can guide researchers when determining the feasibility and limitations of using porcine samples for research on the ACL and knee joint.

## Data availability statement

The original contributions presented in the study are included in the article/supplementary material, further inquiries can be directed to the corresponding author/s.

## Ethics statement

The animal study was reviewed and approved by Institutional Animal Care and Use Committee, Shanghai Jiao Tong University.

## Author contributions

QS performed the dissection experiments, data measurement and the statistical analysis, and wrote the first draft of the manuscript. HW contributed to conception and design of the study, performed the dissection experiments, and revised the manuscript. KH and MT performed data measurement and the statistical analysis. C-KC contributed to conception and design of the study. All authors contributed to manuscript revision, read, and approved the submitted version.

## Funding

This work was supported by the National Natural Science Foundation of China (Grant No. 32101050), the China Postdoctoral Science Foundation (Grant No. 2021M702129) and the SJTU Global Strategic Partnership Fund (2021 SJTU-UoM).

## Conflict of interest

The authors declare that the research was conducted in the absence of any commercial or financial relationships that could be construed as a potential conflict of interest.

## Publisher's note

All claims expressed in this article are solely those of the authors and do not necessarily represent those of their affiliated organizations, or those of the publisher, the editors and the reviewers. Any product that may be evaluated in this article, or claim that may be made by its manufacturer, is not guaranteed or endorsed by the publisher.
